# Importance of Type and Direction of Eyelid Sliding to Mimic Blinking In Vitro for Dry Eye Disease and Contact Lens Research

**DOI:** 10.1167/iovs.67.4.56

**Published:** 2026-04-24

**Authors:** Yong Chen, Hans J. Kaper, Theo van Kooten, Prashant Kumar Sharma

**Affiliations:** 1 Biomaterials and Biomedical Technology, University Medical Centrum Groningen and University of Groningen, Groningen, The Netherlands

**Keywords:** ocular tribology, pendulum friction device, coefficient of friction, contact lenses, dry eye disease

## Abstract

**Purpose:**

Establish a physiologically relevant in vitro ocular friction mode that simulates the natural curvature of the eyelid over the eyeball to investigate the friction mechanisms underlying blink-related discomfort due to contact lens wear (CLW) and dry eye syndrome (DED).

**Methods:**

This study developed a pendulum friction device integrated with the UMT-3 TriboLab to simulate the natural curvature-following motion of the eyelid along the eyeball and measured the kinetic and static coefficients of friction (µ_k_ and µ_s_, respectively) using fresh porcine eyelids and eyeballs, along with soft contact lenses (CLs) as tribo-pairs, under controlled speed, load, and sliding modes. Also, a relatively new parameter, relief period (RP), was used to compare different sliding motions and the presence of CL.

**Results:**

Tribological tests demonstrated that the natural swing motion consistently produced higher µ_k_ and longer RP values compared with artificial sliding or swing, underscoring the importance of sliding direction and mimicking natural blink. Natural swing had significantly higher µ_s_ and µ_k_ than the artificial sliding mode in a low-modulus CL, and it showed significantly higher µ_s_ than the other two artificial modes in a high-modulus CL. No significant difference in RP was observed for either CL.

**Conclusions:**

Ocular tribology is strongly influenced by the direction of sliding. Natural swing motion that mimics blinking produced higher µ_k_ and longer RP than simplified sliding modes. These effects were more pronounced for a low-modulus lens. Accurately replicating the sliding direction is essential for realistic modeling of blinking-related friction.

With global population aging, the prevalence of ocular surface-affecting diseases is increasing, such as dry eye disease (DED) and myopia.^1^^–^^3^ Increased burden of ocular symptoms has increased the demand for contact lenses (CLs) and artificial tears. Inadequate lens–ocular compatibility may increase lens–eyelid friction and cause CL discomfort.^4^ The coexistence of contact lens wear (CLW) and DED complicates the situation even further by aggravating DED symptoms through destabilization of the tear film and increased friction.^5^^,^^6^

Thus, understanding the biotribological characteristics of the sliding ocular interface during blinking under normal and dry eye conditions is essential for the proper design of CLs and artificial tears. Over the past two decades, a wide range of friction measurement methods has been used to evaluate the lubrication properties of hydrating biomacromolecules aimed at dry eye disease and CL to understand the growing discomfort at the end of the day.^7^^–^^14^

Ocular lubrication during blinking is a complex biological system involving sliding interactions between the eyeball and eyelids. The eyelid moves over the cornea in reciprocating sliding with movement speeds reaching 134 ± 4 mm/s.^15^ The lid-wiper displaces the tear film and generates a fluid shear force that induces rapid shear-thinning of the non-Newtonian tear film to reduce friction.^16^^,^^17^ Notably, the contact zone on the eyelids remains static, whereas the contact zone on the cornea surface rapidly moves during blinking. Furthermore, the eyelid follows the curvature of the eyeball during blinking (Fig. 1).

**Figure 1.**
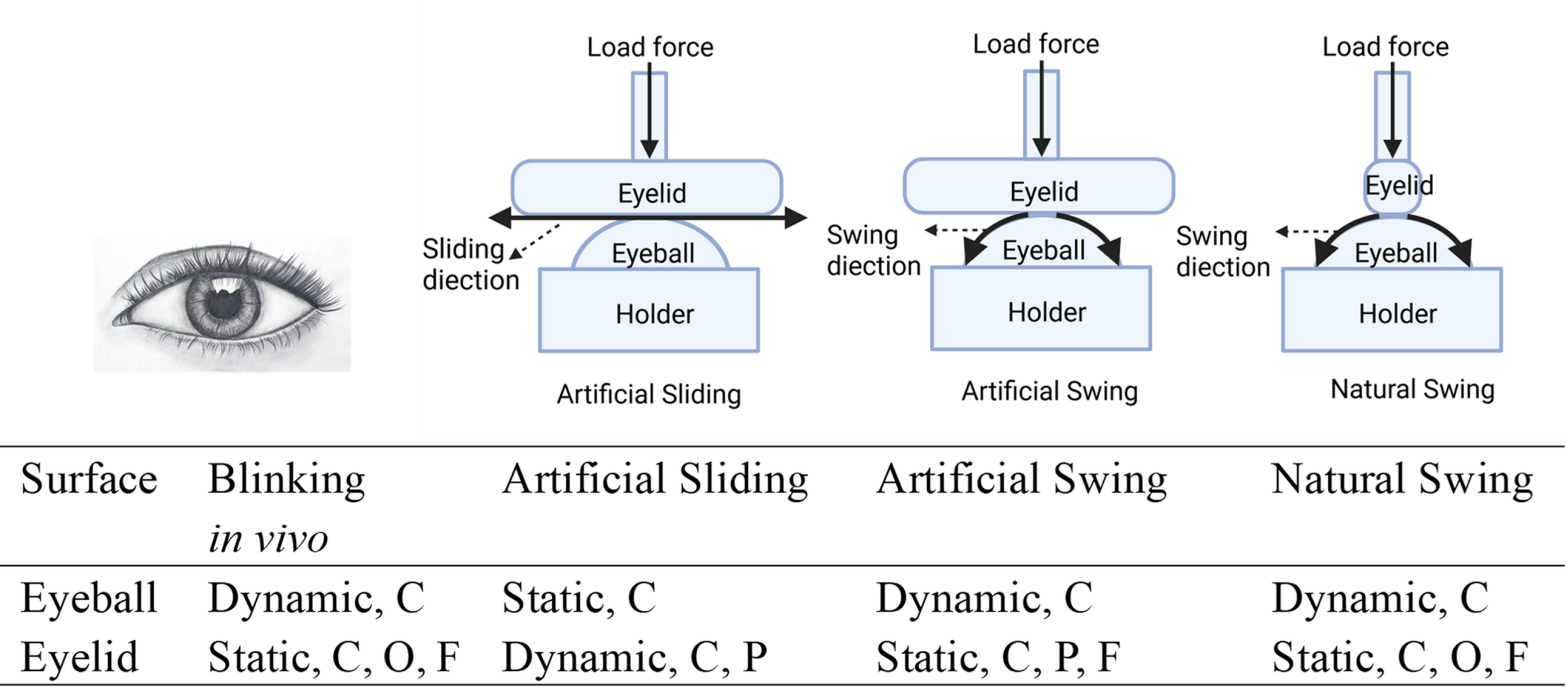
Nature of the contact zone on the two sliding surfaces during blinking in vivo and different in vitro sliding modes (i.e., artificial sliding, artificial swing, and natural swing). When the contact zone remains stationary, it is called “static,” and when it is mobile, it is called “dynamic.” The movement orthogonal to the eyelid is designated with “O,” and the movement parallel is designated with “P.” If the eyelid followed the curvature of the eyeball, then it was designated as “F.” If the contact area remained constant on the surface, then that was designated with “C.”

Mimicking blinking in vitro on a tribometer is a challenge because of high blinking speeds, which need to be achieved in reciprocating sliding within a sliding distance of about 10 ± 1 mm (upper eyelid displacement obtained by geometric inference of palpebral fissure height values of modern populations based on high-speed image measurement),^18^^–^^20^ requiring explosive acceleration and deceleration in short distances. Second, in our previous study, we demonstrated the importance of using fresh eyeball–eyelid pairs for the tribo-pair in DED.^21^ The third difficulty is to maintain the relevant sliding direction, such that the contact zone movement mimics blinking in vivo (Fig. 1). The fourth challenge, which is linked to the first challenge, is to mimic tear fluid and maintain a tear fluid film at the sliding interface.

Previous studies have employed a wide range of tribo-pairs, sliding speeds, and sliding directions.^21^ Often, for simplicity, artificial materials (i.e., nonphysiological friction pairs like stainless steel versus silicone-based hydrogel,^22^ silicon wafer versus polydimethylsiloxane [PDMS] pin,^23^ glass plate versus PDMS^24^^,^^25^) have been used. In our opinion, these are the farthest away from mimicking blinking. Some studies come closer to reality by introducing one fresh tissue (e.g., PDMS versus cornea and glass versus cornea).^8^^,^^14^^,^^26^^–^^28^ Some studies have even used physiological friction pairs,^29^^,^^30^ but maintaining the other conditions shown in Figure 1 remains challenging.

Although nonphysiological tribo-pairs or friction parameters facilitate experimental investigation of ocular tribology, the simplified friction system may obscure the contribution of the ocular surface structure, thereby limiting mechanistic interpretation. And since these pairs do not reflect the physiological environment, the application of design parameters to improve optical aids may carry potential risks for clinical use.

The aim of this study was to evaluate whether modifying the sliding direction to follow the curvature of the eyeball more accurately mimics blinking in vitro. To capture the tribological behavior on the interface between eyelid and eyeball, a pendulum friction device was designed and applied to the pig's eyelids and eyeballs as tribo-pairs. The device is compatible with conventional tribometers; it was integrated with Universal Mechanical Tester (UMT)–3 TriboLab instruments in this study. The forward and backward movement of the UMT-3 sliding module is converted into a circular swing of the device through gear transmission. The sensor obtains the coefficient of friction (µ) of the friction system by monitoring the lateral (tangential) force and applied normal force (*F*_z_) on the contact interface between the eyeball and the eyelid. In this study, µ was compared among natural swing, artificial swing, and artificial sliding under varying *F*_z_ and velocity conditions to illustrate the biomechanical properties of the eyelid–eyeball interface. This study introduces a new parameter, relief period (RP), which is relevant for DED research (Supplementary Fig. S1). In addition, different CL moduli were tested in this study to evaluate the reliability of this measurement by comparing results with previous research. This study, introducing the pendulum friction device, is expected to fill a gap in ocular tribology research and to enhance our understanding of the biomechanical properties of both the eyelid–eyeball and eyelid–CL sliding interfaces.

## Materials and Methods

### Sample Preparation

Fresh porcine eyes were obtained from the local slaughterhouse (Kroon BV, Groningen, the Netherlands) as a by-product of the food industry and transported on ice for immediate use. No animals were sacrificed specifically for this study. All experimental procedures complied with the ARVO Statement for the Use of Animals in Ophthalmic and Vision Research.

Eyelids and eyeballs were processed with reference to previous work.^21^ The eyelid was divided into strips measuring 3 mm (width) × 10 mm (length) using a surgical scalpel and subsequently affixed to a silicone rubber beam with metal pins. After excess muscle tissue from the eyeball was removed, it was affixed to a circular support with metal pins. Subsequently, the eyelid and eyeball were attached to a pendulum friction testing apparatus in accordance with the experimental requirements.

The CL with Young's modulus of 0.3 MPa (1-Day Acuvue Moist; Johnson & Johnson Vision Care, Jacksonville, FL, USA) and 1.5 MPa (Air Optix Night&Day Aqua; Alcon Laboratories, Fort Worth, TX, USA), respectively, were purchased over the counter in Groningen, The Netherlands. Detailed information on CLs is provided in Supplementary Information S3 (Supplementary Table S1). The CLs were taken out of the storage solution, soaked in PBS for 10 minutes, and then placed on the eyeballs with the eyelids closed to allow tear protein absorption for 10 minutes before friction measurements.

### Friction Test SetUp

**Figure 2.**
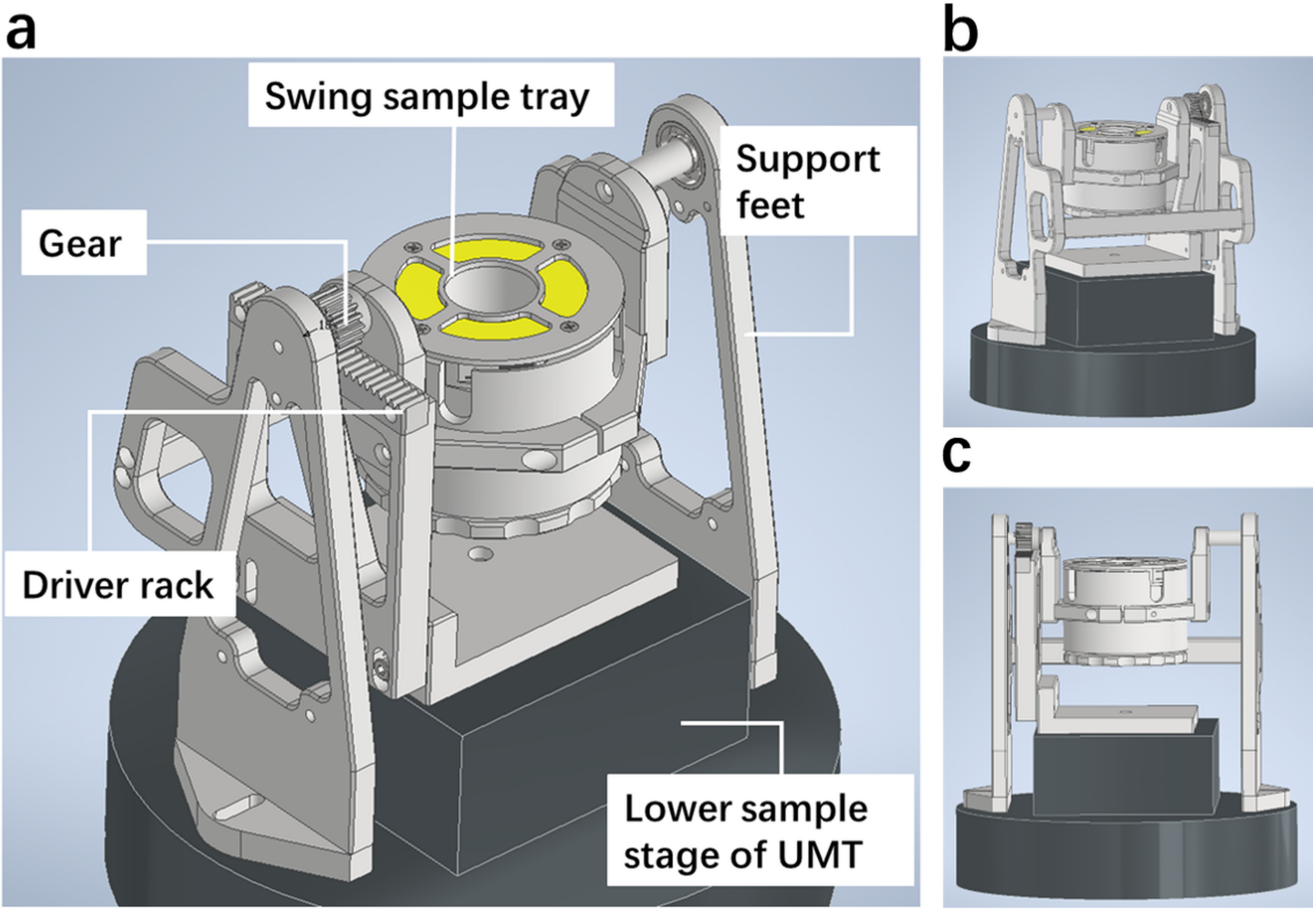
Different views of the pendulum friction device, which can be mounted to any tribometer, including the UMT-3, used in this study Supplementary Movie. (**a**) The top view of components for device. (**b**) The front view of components for device. (**c**) The rear view of components for device.

Friction was measured on a universal mechanical tester (UMT-3; Bruker, Billerica, MA, USA). This tribometer allows multiple mounting possibilities in the reciprocating sliding mode of friction measurement. Figure 1 shows the different sliding types and directions used in this study. “Artificial sliding” (Fig. 1) was used to mimic the most common sliding mode found in literature. “Natural swing” (Fig. 1) mimicked blinking in the best possible way, taking care of whether the contact zone was static or dynamic or the eyelid was sliding in an orthogonal or parallel fashion. “Artificial swing” was used as a control for “natural swing.” To enable natural blinking (Fig. 1) between the eyelid and the eyeball, a pendulum friction device was designed, fabricated at the university medical center groningen machine shop (Fig. 2), and tested for its basic dynamic characteristics and normal force stability. Results are presented in Supplementary Information S3.

The ratio of the measured lateral force (*F*_x_) and the applied *F*_z_ was taken as the coefficient of friction, µ. After the tribo-pair contacted and reached the set value of *F*_z_, stable contact was maintained for 30 seconds before conducting the friction test. The static coefficient of friction, µ_s_, which represented the friction between two nonmoving surfaces, was defined by the maximum µ of the first cycle before the start of movement. Once the sliding starts, Supplementary Figure S1 shows the µ data plotted against sliding time. Fluctuating µ was observed at the beginning, which was due to the establishment of a lubricating film at the sliding interface. Thus, µ from the first 60 cycles was disregarded. The average of the next 60 cycles (120–240 seconds) was taken as the starting kinetic coefficient of friction (µ_k_). Another important parameter used for comparison was the RP, shown in Supplementary Figure S1, and is taken as the number of blinks for which the µ remains low in the presence of a very limited fluid volume as a lubricant placed at the sliding interface. In the present study, we took this fluid volume to be 20 µL.^1^ The contact surface characteristic information of the three models during motion is shown in Figure 1. The RP should be understood as an indicative parameter of the duration for which a drop of 20 µL of lubricant fluid can keep the eyelid–cornea interface lubricated and hydrated. By changing the composition of the lubricant, the RP can be increased, which will be beneficial for patients with dry eye. If they use this fluid lubricant, it can help relieve ocular dryness for a longer duration.

### Statistical Analysis

Statistical analysis was performed on the µ values obtained by the pendulum test method (including natural swing and artificial swing modes) and the traditional method to evaluate the reliability and repeatability of the pendulum friction device. Data are presented as mean ± standard deviation (SD). The sample size (*n*) for each group is indicated in the corresponding figures. Each experiment was repeated independently, and the replicates represent biological replicates unless otherwise specified. Normality of the data distribution was assessed prior to parametric analysis. For comparisons among multiple groups, one-way analysis of variance was performed using Origin2025 software (OriginLab, Northampton, Massachusetts, USA), followed by Tukey honestly significant difference post hoc analysis to determine whether there were significant differences between the µ and RP values obtained by different test modes and CL. Statistical significance was set at *P < 0.05, **P < 0.01, ***P < 0.001, **** *P* < 0.0001. To evaluate the effects of swing velocity and *F*_z_ on µ (Supplementary Fig. S4), the Wilcoxon signed-rank test was employed for paired nonparametric statistical analysis. Effect sizes (*r*) and corresponding 95% confidence intervals were calculated and are reported in Supplementary Information S7.

## Results

### Influence of the Type of Sliding on Eyelid–Eyeball Coefficient of Friction (µ) and RP

Before delving into the details of the results, one must realize that artificial sliding is the most commonly used sliding mode (Fig. 1; Table), and natural swing is the mode that mimics the in vivo blink the closest.

**Table.** Literature Overview of the Reported Coefficient of Frictions for Various Tribo-pairs and Tribological Parameters

Tribo-Pairs	Sliding Type	Sliding Speeds	Sliding Distance	Reference
Blinking in vivo: human eyelid vs. human cornea	Reciprocating sliding	134 ± 4 mm/s (closing phase)26 ± 2 mm/s (opening phase)	9.83 ± 0.17 mm	15,31,32
Human eyelid vs. rabbit cornea	Rotational unidirectional sliding	0.3, 1, 10, 30 mm/s	—	33
Mucin-coated glass vs. human cornea	Reciprocating sliding	0.1 mm/s	1 mm	34
PDMS vs. human cornea	Rotational sliding	0.3–30 mm/s	—	27
Microscope glass specimen vs. pig cornea	Reciprocating sliding	0.5 mm/s	5 mm	35
Glass ball vs. mouse cornea (in vivo)	Reciprocating sliding	250 µm/s	250 µm	36
Hydrogel eyelid vs. engineered ocular surface	Rotational sliding	1, 10 mm/s	—	37
Human corneal epithelial cells vs. CL	Reciprocating sliding	120 mm/s	18.5 mm	38
Conjunctival epithelial vs. immortalized human corneal epithelial cells	Live cell rheometry	\	—	30
Glass disk vs. CL	Reciprocating sliding	0.1, 1, 10 mm/s	1 mm	8

The static, µ_s_, and kinetic, µ_k_, coefficients of friction and RP were compared among the three sliding modes under the *F*_z_ of 30 mN and sliding speed of 8 mm/s. The µ_s_ indicates the amount of lateral force necessary to initiate sliding and is controlled by the adhesive interactions between the two surfaces at a sliding speed of 0 (i.e., at the start and end of the blink). The µ_k_, on the other hand, indicates the lateral force required to maintain sliding motion. The RP shown in Supplementary Figure S1 is the number of blinks for which the µ_k_ remains low in the presence of 20 µL of fluid lubricant.^21^

**Figure 3.**
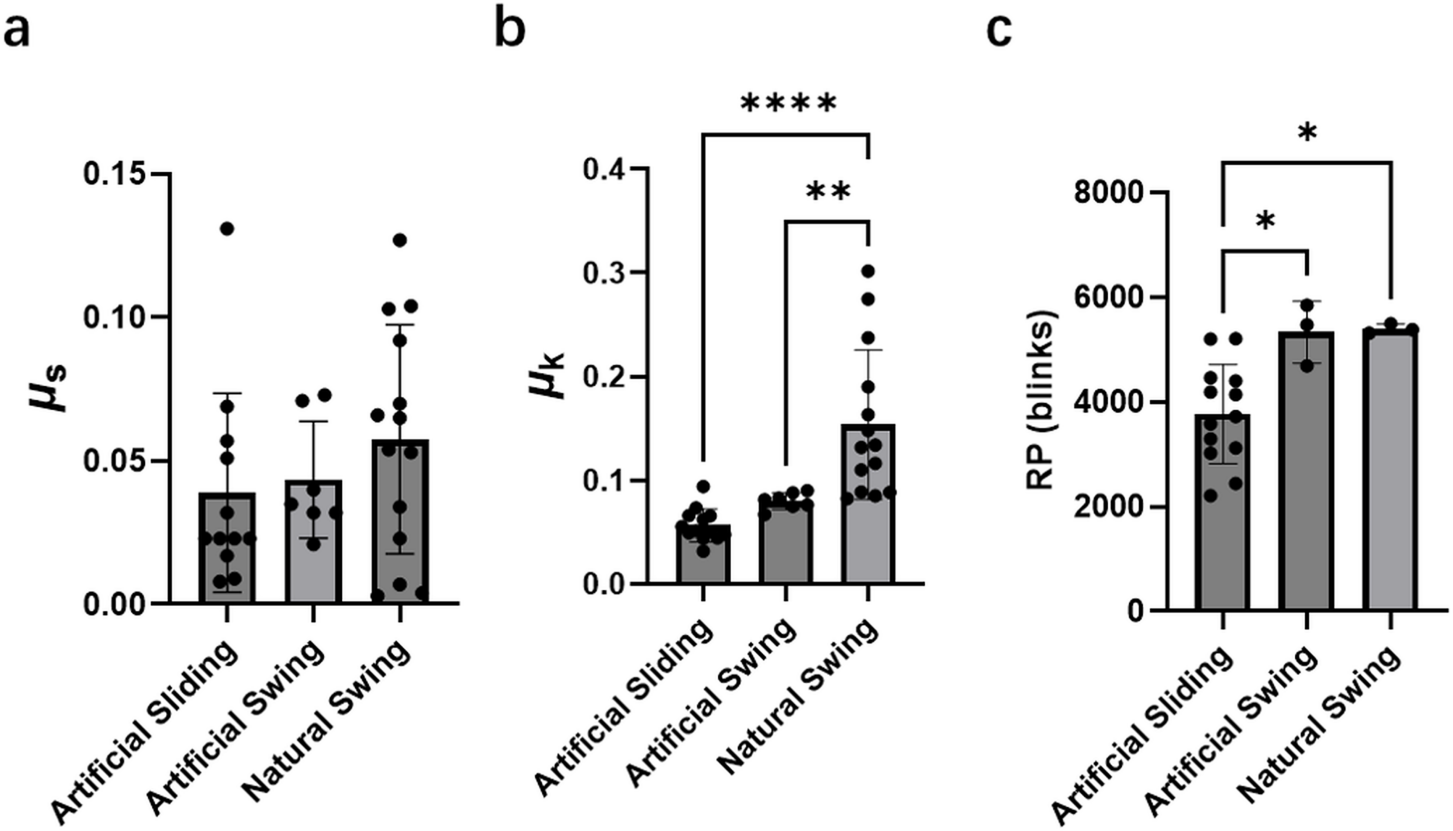
The µ_s_ (**a**), µ_k_ (**b**), and RP (**c**) between eyelid and eyeball, and the effect of sliding type at an 8-mm/s speed when *F*_z_ is set as 30 mN. Data are presented as mean ± SD with individual data points shown. For µ_s_ and µ_k_ measurements: artificial sliding (*n* = 12), artificial swing (*n* = 7), and natural swing (*n* = 14). For RP measurements: artificial sliding (*n* = 13), artificial swing (*n* = 3), and natural swing (*n* = 3).

As shown in Figure 3a, the µ_s_ of the artificial swing and natural swing modes were 0.043 ± 0.020 and 0.058 ± 0.042, respectively, and were not significantly different from that measured under the artificial sliding mode (0.039 ± 0.035).

The µ_k_ value obtained under the natural swing mode was 0.15 ± 0.069, which was twofold higher than artificial swing (0.080 ± 0.0073) and threefold higher than artificial sliding (0.057 ± 0.015) modes, as shown in Figure 3b. Similarly, the RP under natural swing mode was 5398 ± 90 blinks, which was significantly higher than that measured under the artificial sliding mode (3771 ± 946). Thus, these results indicate that the friction type and direction strongly influence both µ_k_ and RP.

### Influence of the Type of Sliding on Eyelid–Contact Lens Coefficient of Friction (µ)

To explore the effect of placing a CL on the cornea surface, giving rise to the eyelid–CL sliding interface, the µ_s_, µ_k_, and RP were measured on both low-modulus (0.3 MPa) and high-modulus (1.5 MPa) CLs. The eyelid and cornea are highly deformable soft tissues with Young's modulus of 0.1 and 0.4 MPa,^21^ respectively, highlighting the compliant nature of the ocular surface and its interaction with the CL. Each lens was used only once. The friction tests consisted of 120 sliding cycles per measurement, with a total duration of no more than 5 minutes to limit dehydration of soft lenses. For RP parameter testing, measurements were intentionally extended until lens dehydration, identified by a sharp rise in the friction coefficient, to evaluate moisture retention behavior.

**Figure 4.**
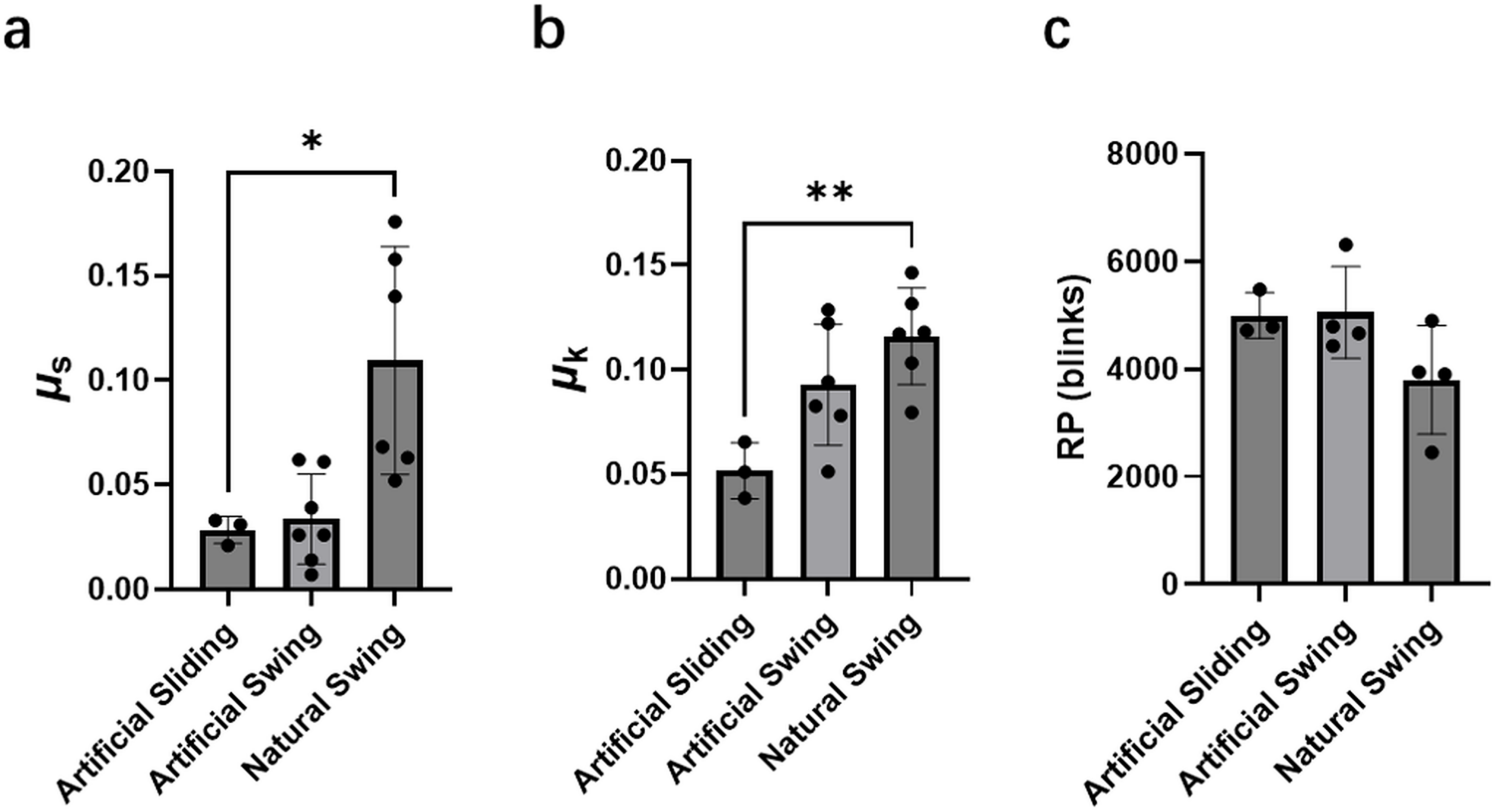
The µ_s_ (**a**), µ_k_ (**b**), and RP (**c**) between eyelid and low-modulus CL and the effect of the type of sliding at an 8-mm/s speed when the *F*_z_ is set as 30 mN. Data are presented as mean ± SD with individual data points shown. For µ_s_ and µ_k_ measurements: artificial sliding (*n* = 3), artificial swing (*n* = 7), and natural swing (*n* = 6). For RP measurements: artificial sliding (*n* = 3), artificial swing (*n* = 4), and natural swing (*n* = 3).

For low-modulus CL (Fig. 4a), the µ_s_ value obtained under the natural swing mode (0.11 ± 0.055) was ∼4-fold higher than that measured under the artificial sliding (0.028 ± 0.0064). In contrast, the µ_k_ (Fig. 4b) value doubled from 0.052 ± 0.013 under the artificial sliding mode to 0.12 ± 0.023 under the natural swing mode, showing a statistically significant difference (*P* < 0.01). The RP results (Fig. 4c) indicated that the RP under the natural swing mode was a lower mode than the other two modes, with no significant difference.

**Figure 5.**
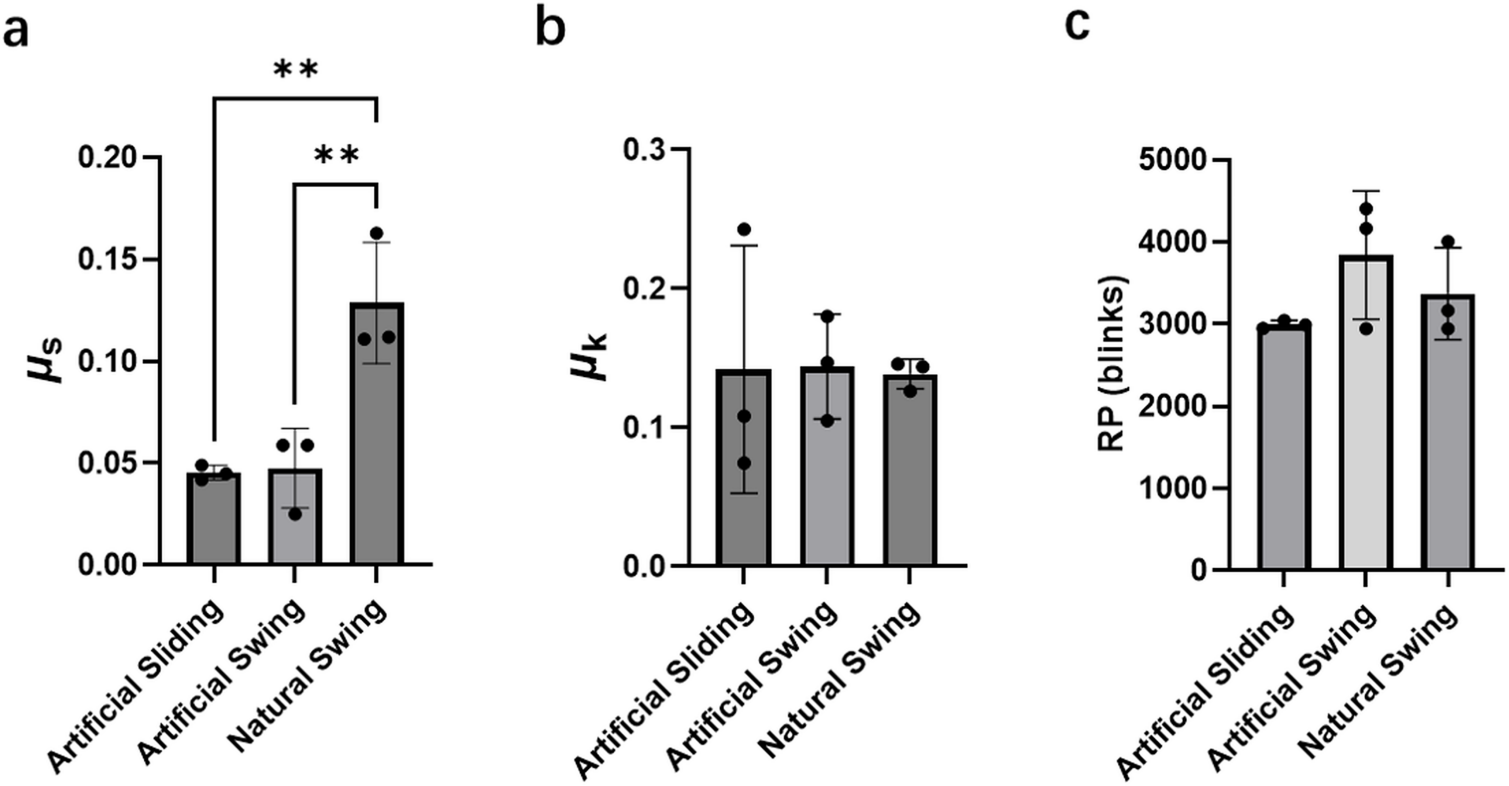
The µ_s_ (**a**), µ_k_ (**b**), and RP (**c**) between eyelid and high-modulus CL and the effect of the type of sliding at an 8-mm/s speed when the *F*_z_ is set as 30 mN. Data are presented as mean ± SD with individual data points shown. *n* = 3 independent experiments per group.

For the high-modulus CL, the µ_s_ (Fig. 5a) under the natural swing mode (0.13 ± 0.030) was more than double that measured under the two artificial modes (artificial sliding, 0.045 ± 0.0035; artificial swing, 0.048 ± 0.020). In contrast, the values of µ_k_ for the three sliding modes on high-modulus CL surfaces were very similar, with all values being approximately 0.14 (Fig. 5b). The RP values obtained were between 3000 and 4000 blinks, with no significant difference between modes (Fig. 5c).

## Discussion

In our previous work, we demonstrated the importance of using fresh ocular tissues as tribo-pairs in experiments relevant to dry eye disease research.^21^ The present study extends this understanding by showing that sliding direction relative to the eyelid and alignment with corneal curvature are equally critical parameters in ocular tribology. In this study, fresh ocular tissue was employed as a tribo-pair and tested with a pendulum-type friction device compatible with the UMT-3 system, which allows the eyelid to slide along the curvature of the eyeball and thereby more faithfully simulates the physiological blinking.

### Physiological Relevance of Sliding Speeds

The maximum physiological blink speed has been estimated at ∼134 mm/s.^15^ However, such velocities are difficult to achieve with a reciprocating sliding mode (Table) because of acceleration limitations. Most in vitro studies employ sliding speeds of 0.1 to 30 mm/s.^8^^–^^10^^,^^39^^–^^41^ While torsional and oscillatory devices can reach higher speeds (90–200 mm/s),^13^^,^^14^^,^^42^ these alternative devices cannot accommodate intact ocular tissues or reproduce eyelid motion along the corneal curvature, and in some cases, the µ is only indirectly inferred.^13^ For reasons of experimental operability, speeds between 0.1 and 10 mm/s remain most widely adopted. In line with this, we selected a range of 1 to 9.8 mm/s for testing in this study. As can be seen from Supplementary Table S3, the Stribeck number (also called the Hersey number) for our sliding experiment (8.2 × 10^−9^) was 16-fold lower than natural blinking (1.3 × 10^−7^) in vivo, strongly indicating that our experiments remained in the boundary lubrication regime.

At low speeds (i.e., at the start and the end of a blink), the tear film is very thin, and lubrication is primarily handled by mucin brushes on the cornea and conjunctival surfaces. In this case, friction is mainly determined by the steric hindrance and electrostatic repulsion between the opposing polymeric brushes.^17^ When brush density decreases, interleaving occurs, leading to increased µ_s_ and µ_k_. This behavior reproduces in the natural swing mode. Therefore, differences µ in the interlens and direction-dependent tribological behavior remain effective in the boundary lubrication state.

As speeds approach the physiological blink rate, the system transitions to mixed or hydrodynamic lubrication,^43^ where tear film load support plays a crucial role. In the tear film layer, the surfaces are separated, friction becomes more dependent on the tear film's viscosity and fluid properties, the influence of surface microstructure may decrease, and direction-dependent effects may weaken.

For RP, the main influencing factors are the tear film volume, spreading efficiency, and surface wettability. These factors are not strongly dependent on the lubrication stage; therefore, the trend of RP variation is expected to remain unchanged. It is worth noting that shifting the system toward hydrodynamic lubrication by increasing speed increases blink frequency, thereby increasing the probability of tear expulsion and accelerating evaporation in in vitro friction experiments. This may widen the difference between the artificial and natural swing groups but will not alter the overall trend.

### Eyelid–Cornea Interface Morphology Helps Understand the Differences in Friction and RP Results From Different Modes

Since both the cornea and eyelid surfaces contain the mucin and proteoglycan 4 (PRG4) containing glycocalyx,^27^^,^^33^^,^^44^ adhesion at the eyelid–cornea interface is limited at the start or end of the sliding cycle, which may explain the similar µ_s_ values across modes.

**Figure 6.**
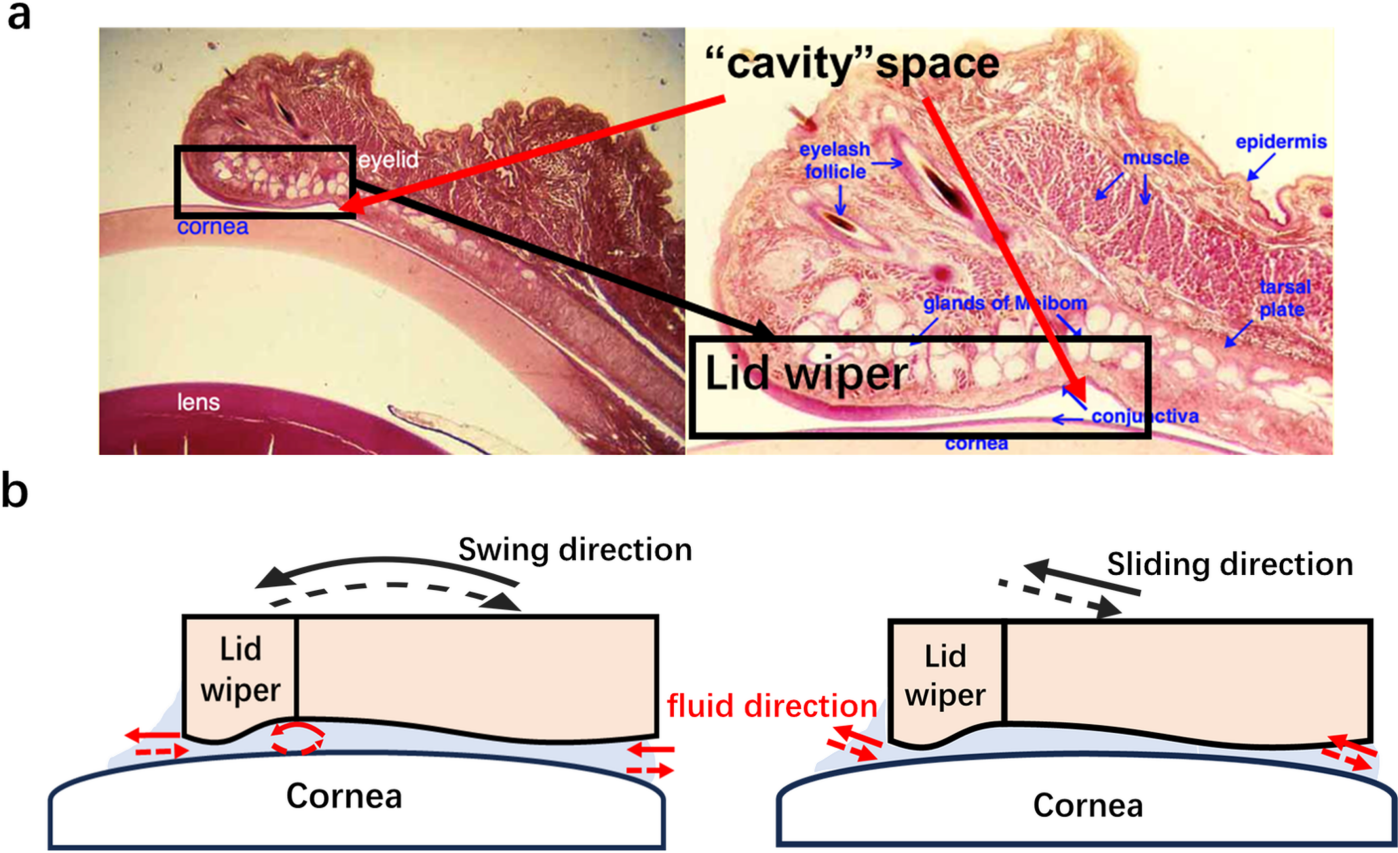
(**a**) Histologic section of eyelid and cornea tissue (reprinted and adapted with permission from David King, SIU School of Medicine, https://histology.siu.edu/ssb/EE018b.htm and https://histology.siu.edu/ssb/EE008b.htm). (**b**) Fluid flow behavior in natural swing (*left*) and artificial sliding (*right*) modes.

A significant difference in µ_k_ was observed between the natural swing and artificial sliding modes at the eyelid–cornea (eyeball) interface. The higher µ_k_ in the natural swing mode can be attributed to the lid wiper structure, which concentrates stress at the anterior eyelid margin. This localized high pressure induces microstructural deformation of the corneal and eyelid surfaces, thereby elevating resistance during blinking.^45^^–^^47^ Furthermore, in natural swing mode, the lid wiper can perform its role as a wiper smearing the lubricant film on the eyeball (both cornea and conjunctiva) surface but has a higher µ_k_ in this process. RP analysis further highlighted the role of eyelid morphology. RP values in the natural swinging mode were significantly longer than those in linear sliding, suggesting that cyclic redistribution of lubricant and the formation of a local lubricant retention zone in the cavity (Fig. 6) prolong tear film availability and slow drying.

In contrast, in the artificial sliding mode, the lid wiper cannot effectively redistribute the lubricant film. Although the cavity may transiently supply fluid to the contact zone, it also promotes unidirectional lubricant loss, impairs film recovery, and increases local rupture and drying,^48^^,^^49^ resulting in lower RP. These findings underscore the functional importance of eyelid structure in maintaining lubrication and illustrate how inappropriate simulation of blinking (i.e., artificial sliding mode) may obscure this role.

### Insertion of Contact Lens at the Eyelid–Eyeball Interface

To mimic CLW in our system, we placed two types of soft CLs in the intact eyelid and the eyeball for 10 minutes, then mounted them on the friction device to initiate sliding in the three modes shown in Figure 1. In this case, we observed major sliding between the eyelid and the CL surface. The influence of friction direction was particularly evident in eyelid–CL tribo-pairs. The µ_s_ values of the natural swing mode were significantly higher than those of the artificial sliding and swing modes, indicating that directional effects are more pronounced in the eyelid–CL system. In contrast to the eyelid–eyeball system, the CL surface contains far fewer tear proteins than the healthy cornea surface, which contains a thick glycocalyx. The CL surface does contain some tear proteins because we placed the CL between the intact eyelid and eyeball for 10 minutes, but this layer is far too ineffective to avoid the adhesive interactions manifesting themselves as µ_s_, especially in the natural swing mode. Furthermore, the heightened µ_s_ values observed at the eyelid–CL interface as compared to the eyelid–cornea interface could explain the end-of-day discomfort experienced by CL wearers mode.^8^

Material modulus, together with lens surface chemistry and hydration characteristics, further modulated tribological behavior. Direction-dependent effects on µ_k_ were mainly observed in low-modulus lenses, whereas high-modulus CL (1.5 MPa) exhibited reduced sensitivity. The stiffness of the low-modulus CL (0.3 MPa) closely approximates that of the cornea (0.1 ± 0.08 MPa) and eyelid (0.4 ± 0.2 MPa),^21^ allowing compliant tissue-to-tissue contact. In contrast, high-modulus (1.5 MPa) lenses deform the eyelid interface, while interfacial behavior is further influenced by their distinct surface chemistry and lower equilibrium water content. Taken together, these observations suggest that CL lubricity is governed by the coupled effects of tribo-pair mechanical compliance, surface physicochemical properties, and contact geometry, rather than elastic modulus alone. As the two lenses differ in both elastic modulus and surface chemistry, these factors cannot be fully decoupled but highlight their combined influence under physiologically relevant sliding conditions.

### Limitations and Future Perspectives

Despite these insights, the study has limitations. The pendulum device reached only ∼10 mm/s, well below physiological blink velocities. Furthermore, PBS was used as the lubricant rather than human tear fluid. Therefore, the present setup precludes investigation of elasto-hydrodynamic lubrication regimes during healthy blink. Nevertheless, because direct evidence of in vivo blinking friction remains unavailable, the proposed tissue friction mode offers an operational and reproducible approach for probing ocular tribology under boundary lubrication conditions.

Our findings hold significant implications for dry eye research, although interpreting previous studies on ocular and CL tribology based on our results remains difficult. This is because the sliding interfaces used are very different, and the parameter RP has not been used before. However, our results suggest that eyelid–CL friction may be more relevant than CL–cornea friction when correlating tribology with end-of-day comfort scores.^8^

Our results suggest that mechanical compliance, surface chemistry, hydration state, and contact geometry are intrinsically coupled at the eyelid–lens/cornea interface. Specifically, studies on the comparison of CL of different compositions, stiffnesses, and surface properties, while sliding against the eyelid, are desirable.

## Conclusions

This study demonstrates that ocular tribology is strongly influenced by sliding direction. Using a pendulum-type friction device, we showed that natural swing motion, which mimics physiological blinking, consistently produced higher µ_k_ values and longer RP than the artificial sliding mode most often used for its simplicity. These effects were particularly pronounced for low-modulus CL, suggesting that material compliance and interfacial deformation may contribute to the observed differences, although other material properties, such as surface wettability and water content, may also play a role.

## Supplementary Material

Supplement 1

Supplement 2
