# Supplementary material for: Importance of Type and Direction of Eyelid Sliding to Mimic Blinking In Vitro for Dry Eye Disease and Contact Lens Research
Source: Invest Ophthalmol Vis Sci. 2026 Apr 24;67(4):56. doi: 10.1167/iovs.67.4.56 (PMC13109897; doi:10.1167/iovs.67.4.56)
Supplement: Supplement 1 [file iovs-67-4-56_s001.pdf]

Supplementary Information

For Investigative Ophthalmology & Visual Science

Importance of type and direction of eyelid sliding to mimic blinking in vitro, for dry eye disease and contact lens research

Yong Chen[1], Hans J. Kaper[1], Theo van Kooten[1], Prashant Kumar Sharma[1]

[1] Biomaterials and Biomedical Technology, University medical centrum Groningen and University of Groningen, Groningen, The Netherlands

Corresponding Author:
Prashant Kumar Sharma
Biomaterials and Biomedical Technology
University medical centrum Groningen and University of Groningen, Groningen
Antonius Deusinglaan-1
Groningen, 9713AV, The Netherlands
p.k.sharma@umcg.nl

Supplementary information 1:

Materials and Methods:

Pendulum friction device:

To perform the swing movement, which allowed the sliding to follow the curvature of the eyeball the 'Pendulum friction device' was designed and fabricated at the Machine Shop of the University Medical Center Groningen as shown in Figure 2.

The pendulum friction device consists of two parts: the drive rack and the swinging sample tray. The drive rack is fixed to the lower sample stage of the UMT-3 system with screws. The support feet of the swinging sample tray are secured to the base of the UMT-3, aligning the gears with the drive rack. The lower sample stage of the UMT-3 converts the UMT-3's linear reciprocating motion into the sample's pendulum-like motion through the rack-and-pinion transmission system. Therefore, the contact surface speed and motion distance of the sample must be calculated from the linear speed. The sliding velocity v_{eye} (mm/s) is equal to the product of the angular velocity ω (rad/s) and the radius r (11 mm) of corneal curvature as shown in Equation (1):

$$v_{eye} = r\omega \qquad (1)$$

The angular velocity of the eyeball is the same as the angular velocity of the gear, so the sliding velocity of the corneal contact surface can be calculated by equation (2):

$$v_{eye} = \frac{rv_{gear}}{R} \qquad (2)$$

In the equation 2, v_{gear} is the sliding speed of gear (which is equivalent to the speed of the drive rack) which is equal to the sliding speed of UMT-3 lower sample stage used, R is the radius of gear that is 9 mm. This makes the $\frac{r}{R}$ ratio to be 1.22 giving rise to v_{eye} to be always

1.22 times higher than the set speed of the lower sample stage, v_{gear}. In this experiment, the maximum linear speed of the UMT-3 linear drive is 10 mm/s, the applied normal load range is 5 to 500 mN, and the resolution is 50 μN.

Two different ways were tested to simulate blinking using the pendulum friction device (Figure 2). When the eyelid was in the anatomically normal position to simulate blinks from up-to-down, this was termed 'Natural Swing'; and when the eyelid was rotated 90 degrees to simulate blinks in a lateral direction from left-to-right, this was termed 'Artificial Swing'. The method of sliding back and forth directly along the long axis of the eyelid is called 'Artificial Sliding'.

Definition of relief period (RP) parameter

In Figure S1, three distinct regions can be identified. The first region corresponds to a low plateau, during which the μ_k remains stable or shows a slight increase, indicating a steady sliding regime. This is followed by the relief stop, characterized by a sharp rise in μ_k. The third region corresponds to the onset of corneal epithelial damage, during which intracellular fluid is released from the epithelial tissue. This released fluid re-lubricates the sliding interface, resulting in a pronounced decrease in μ_k. RP was defined based on the rate of increase in the rate of change of the μ_k. The RP was identified by the rate of increase of the rate of change of μ of at least 0.01 as per equation 1 and shown in Figure S1. If this value was not constant in the subsequent cycles, the value was discarded, and the next values were investigated.

$$RP = Number\ of\ Blink\ till\ \frac{\partial^2 \mu}{\partial^2 t} > 0.01 \qquad (1)$$

In equation (1), μ is the friction coefficient and t is the Number of Cycles.

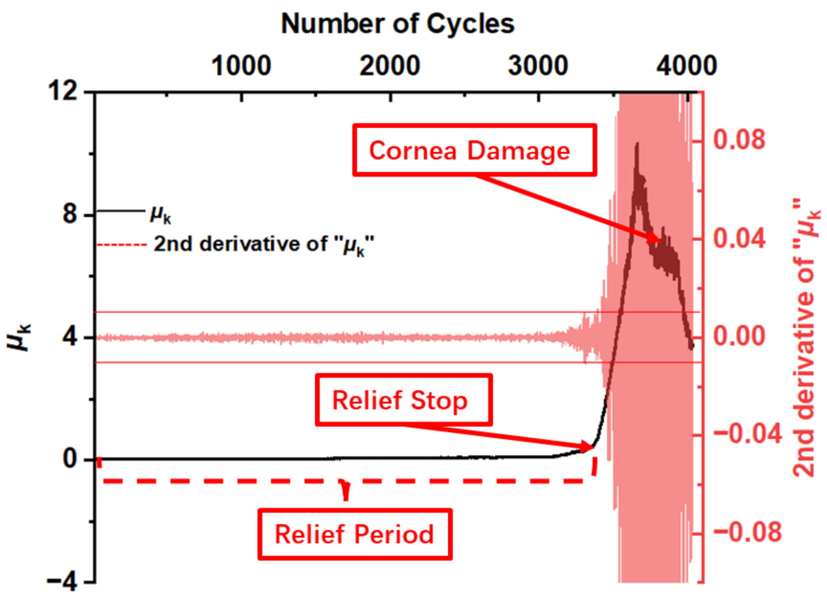

Figure S1. Schematic of Changes in μ with Number of Cycles[1]

Supplementary information 2: Supplementary Movie S1.swing eyeball:

Supplementary Movie S1. swing eyeball.mp4

Supplementary information 3: Information of Contact Lenses

The soft contact lenses used in this study represent two distinct material classes with different physical and chemical properties. The low-modulus lens (1-Day Acuvue Moist, Johnson & Johnson Vision Care) is made of a hydrogel polymer (Etafilcon A) with a high water content (~58 %) and proprietary LACREON® hydration technology, offering high flexibility and surface wetting under physiological conditions. In contrast, the high-modulus lens (Air Optix Night & Day Aqua, Alcon Laboratories) consists of a silicone hydrogel material (Lotrafilcon A) with a low water content (~24 %) and very high oxygen permeability (Dk/t ~175).

Table S1:

Features	1-Day Acuvue Moist	Air Optix Night & Day Aqua
Material Type	Hydrogel (Etafilcon A)	Silicone Hydrogel (Lotrafilcon A)
Water Content	~58%	~24%
Oxygen Permeability (Dk/t)	25.5×10^{-9} (cm·mL O_2)/(s·mL·mmHg)	~175×10^{-9} (cm·mL O_2)/(s·mL·mmHg)
Hardness (Modulus)	0.3 MPa	1.2–1.5 MPa

Supplementary information 3:

Results

1. Dynamic characteristics of the pendulum friction device

During reciprocating sliding on a tribometer, the drive system accelerates from rest to a prescribed sliding velocity, maintain this velocity for a long duration, and decelerates to zero before revering direction. The time duration of each cycle for which the set speed is maintained depends on the acceleration characteristics of the device. To quantitatively characterized this behavior, two key parameters were defined: 'velocity maintenance ratio' per cycle and 'peak acceleration' characteristics within a set sliding distance. Velocity maintenance ratio was defined as the proportion of the cycle time during which the preset sliding velocity was maintained relative to the total cycle time (Figure S2). Peak acceleration was defined as the acceleration demonstrated by the pendulum friction device to achieve the set sliding speed in the set sliding distance (Figure S2). Both parameters are determined by the dynamic performance of the lower sample stage drive of the UMT-3, shown in Figure S1. Figure S2_c demonstrates the relationship between set sliding velocity (1.2, 2.4, 4.9, and 9.8 mm/s) and sliding distances (1.2, 2.4, 4.9, and 9.8 mm) in terms of the velocity maintenance ratio. The 'velocity maintenance ratio' decreased with increasing sliding speed at a set sliding distance, but increased with increasing sliding distance for the set sliding speed. Acceleration profiles

revealed distinct patterns as shown in Figure S2_d. The maximum acceleration used by the device to achieve the set speed demonstrated a strong linear dependence on sliding velocity, with proportionality coefficients varying. Notably, longer sliding distances required proportionally higher accelerations to achieve equivalent target velocities, suggesting distance-dependent inertial effects in the actuation system. A maximum acceleration of 160 ± 33 mm/s^2 was observed for the ‘pendulum friction device’. However, when the set sliding distance was minimal (1.2 mm) and at the highest sliding speed (9.8 mm/s), the acceleration remained limited to 83.5 ± 28.2 mm/s^2. Figure S2_e shows that the total cycle time decreases with increasing speed and decreasing distance. For reference, the sliding distance during an adult blink *in vivo* is about 10 mm(Estimated from the speed and duration of a full blink)[2].

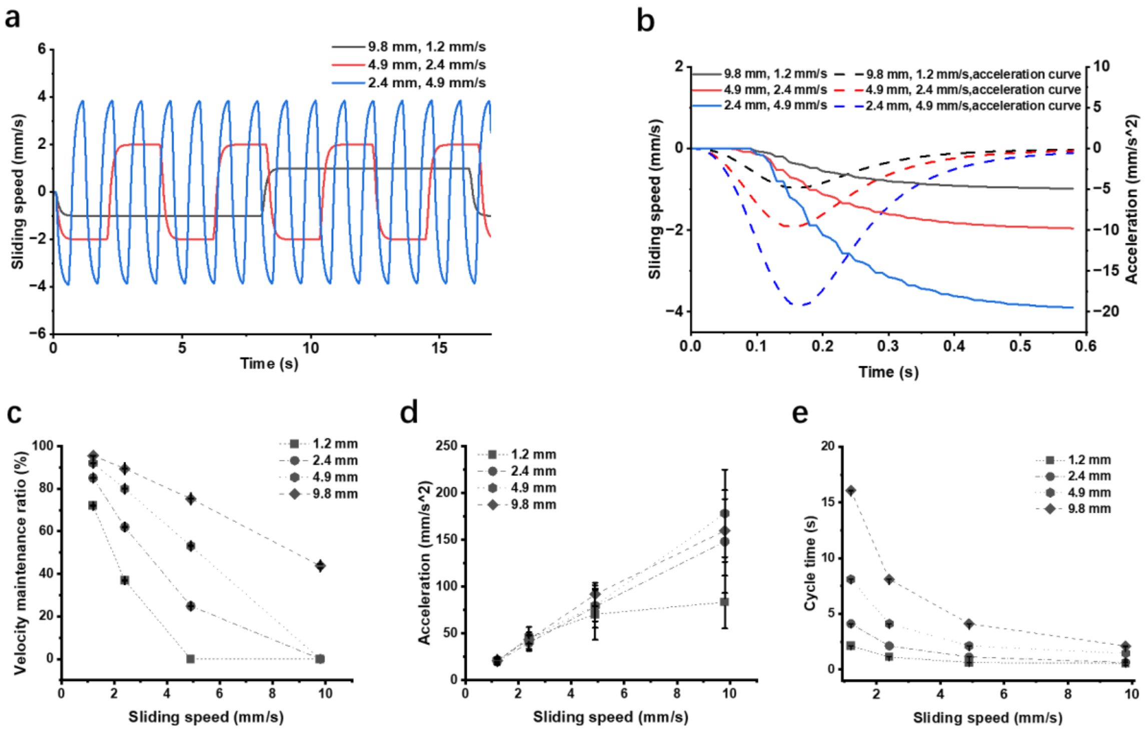

Figure S2. Dynamic characteristics of the pendulum friction device: (a) Schematic illustration of time-dependent variations in sliding speed and (b) acceleration under different conditions; (c) the velocity maintenance ratio; (d) Maximum acceleration demonstrated by the device to attain the set speed for the eyelid-eyeball tribo-pair; (e) Total cycle time.

2. Normal force (F_z) stability.

The measured friction force, F_x and μ not only depend on the sliding speed but also on the normal force F_z. UMT-3 is equipped with an active control mechanism that adjusts the position of the upper carriage to maintain the F_z close to the set value. The ability of UMT-3 to maintain a stable F_z depends on the interplay between the response time set on the device in terms of the sensor's force feedback parameter and the sliding speed. Given that biological tissues were used on both contacting surfaces with either horizontal or swing sliding, it is essential to evaluate the stability of F_z when the pendulum friction device is applied to ocular tribology measurements. To investigate the stability of F_z between the eyelid and the eyeball, a dedicated test bench with a sinusoidal pendulum motion was employed to simulate blinking. The root mean square deviation (RMSD) of F_z was quantified for a range of sliding distances (1.2 mm, 2.4 mm, 4.9 mm, and 9.8 mm) and different sliding speeds (1.2 mm/s, 2.4 mm/s, 4.9 mm/s, and 9.8 mm/s) at a set F_z value of 30 mN. As shown in Figure S3, the RMSD was 10-15% of F_z and shows a slightly increasing trend with increasing speed, but not statistically significant. This result indicates that increasing speed leads to slight fluctuations in F_z, which more likely attributable to the sensor noise changing with speed rather than the actual mechanical response. Therefore, the system remained largely insensitive to changes in sliding speed with respect to the dynamic characteristics of F_z.

When the speed was kept constant, changing the swing amplitude between 2.4 and 9.8 mm leads to a slight decrease in RMSD; however, no significant difference is observed. Notably, the RMSD of F_z is lower at the swing amplitude of 1.2 mm, when the speed was 4.9 or 9.8 mm/s compared with the swing amplitude ranging from 2.4 to 9.8 mm. This apparent reduction is likely attributable to the short strokes, which can approximate the eyeball swing trajectory as planar, and due to limitation of the system that the interface velocity cannot reach the set speed under such a short stroke (velocity maintenance ratio is close to zero, Figure S1_c).

Therefore, such cases need to be carefully accounted for in practical applications. Effect of swing velocity and normal force on Natural Swing model.

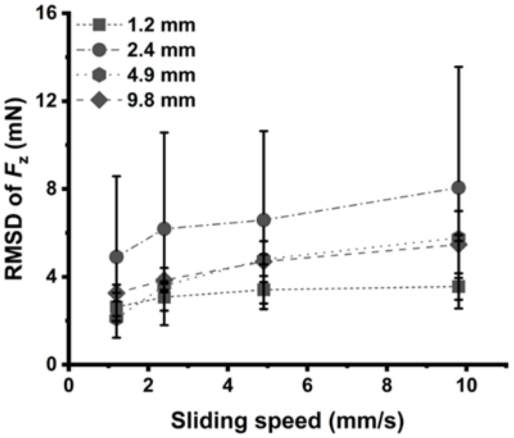

Figure S*3*. F_z stability under different displacements as a function of sliding velocity, when the F_z set value was 30 mN.

The dynamic evaluation of the pendulum friction device confirmed its suitability for simulating reciprocating natural swing motion relevant to ocular tribology. The speed maintenance ratio decreased with increasing sliding speed but increased with greater sliding distance, while peak acceleration showed a strong linear relationship with sliding speed, modulated by sliding distance (Figure S2). At very short distances (e.g., 1.2 mm), the device was limited in its ability to achieve high speeds, resulting from constrained maximum acceleration of about 160 mm/s^2. Importantly, the stability of F_Z across various test conditions, with only minor and statistically insignificant fluctuations, demonstrated the robustness of the device to changes in sliding parameters (Figure S3).

To evaluate the effects of swing velocity and F_z on μ, the Wilcoxon signed-rank test was employed for paired nonparametric statistical analysis. As shown in Figure S4, measurement

data were obtained from the same contact lens sample and test location under each test condition, while systematically varying either the swing velocity or F_z. For each velocity gradient (n=4) and F_z gradient (n=6), tests were repeated on different contact lenses. Specifically, under fixed F_z conditions, the same lens underwent tests at sequential velocities. Correspondingly, under fixed velocity conditions, F_z was systematically varied while repeating the aforementioned procedure.

The results in Figure S4_a show that with the increase of speed, the μ_s measured under the Natural Swing models between eyelid and eyeball exhibited a decreasing trend. In contrast, the μ_k value increased with increasing speed (Figure S4_b). When the speed increased from 1.2 to 4.9 mm/s, the μ_s decreased from 0.057 ± 0.042 to 0.033 ± 0.030. By contrast, the μ_k showed an opposite trend, increasing from 0.10 ± 0.018 to 0.17 ± 0.053. At the speed of 9.8mm/s, increasing F_z resulted in a significant decrease in μ_k as shown in Figure S4_d. The value of μ_s exhibited an increasing trend with increasing F_z, while no significant difference (Figure S4_c). The Stribeck curves are typically used to characterize the lubrication mechanism region. Figure S4_e illustrates the trend of μ_k as a function of $v\eta/P$. Across the investigated range of $v\eta/P$ (10^{-9}–10^{-7}), μ_k remains within a narrow interval of approximately 0.08–0.15. No monotonic increase or decrease in μ_k is observed. Instead, a slight increase in μ_k appears at intermediate $v\eta/P$ values, followed by stabilization at higher values. (Figure S4_e).

Analysis of combined effects of swing speed and load revealed that μ increased systematically with velocity (Figure S4_b), consistent with the trends reported by Tannin et al. [3] for rabbit eyelid–cornea pairs, despite differences in absolute values. This velocity dependence likely reflects the viscoelastic and morphological properties of the tissues. At higher velocities, delayed recovery of deformation increases steric hindrance, raising friction [4, 5]. Under physiological conditions, cornea microvilli form a brush-like layer enriched in mucins, which facilitates boundary lubrication via steric and electrostatic effects, particularly at blink

initiation and termination [6-8]. In dry eye, loss of this mucin-rich structure diminishes hydration and steric exclusion, resulting in elevated friction during blinking [6, 9-15].

Load-dependent tests revealed distinct behaviors in μ_s and μ_k (Figures S4_c and S4_d). Increases in load expanded the contact area (Figure S5), increasing mucin interactions and thereby elevating μ_s [6]. Conversely, average contact pressure decreased with increasing load, consistent with mechanical models of tissue deformation [13].

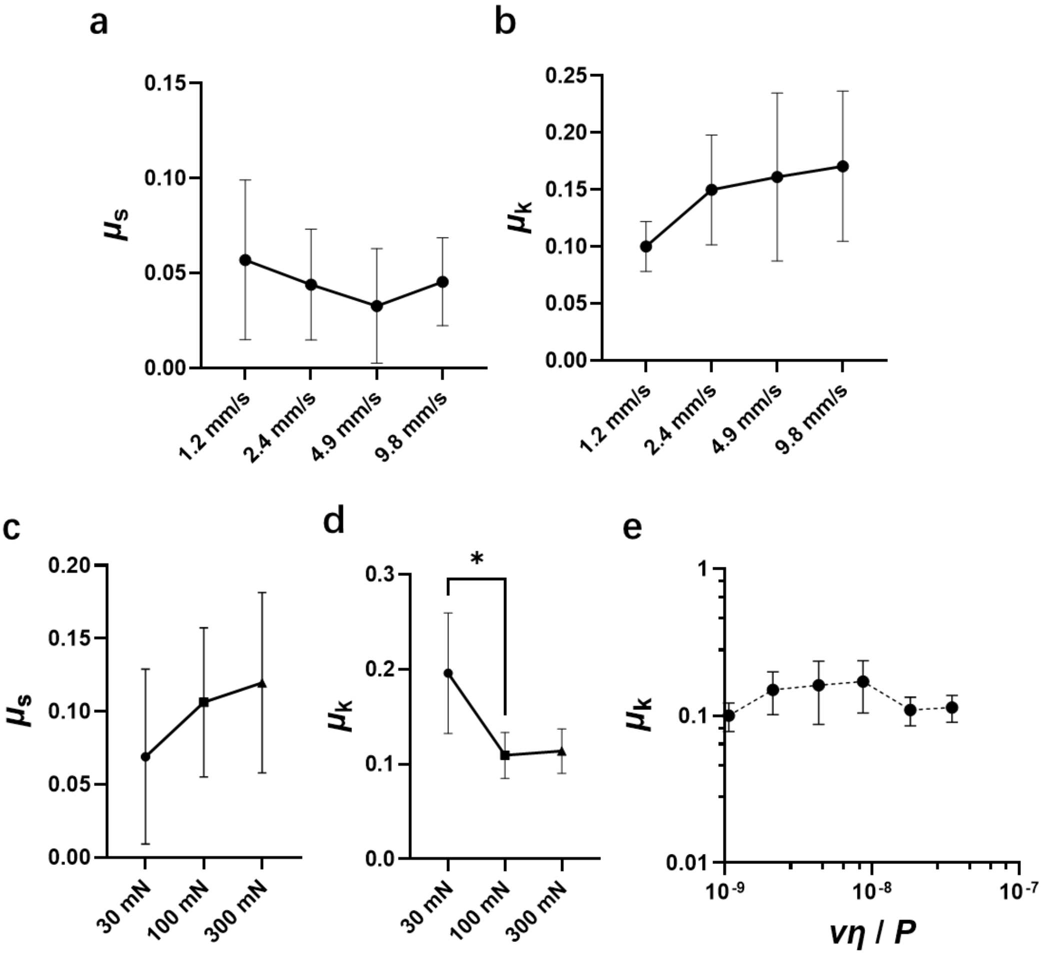

Figure S4. Effect of sliding speed and normal force in Natural Swing, (a) Relationship of μ_s and velocity under 30 mN normal force (n=4); (b) Relationship of μ_k and velocity when the F_z set as 30 mN (n=4); (c) Relationship of μ_s and normal force at 9.8 mm/s (n=6); (d) Relationship

of μ_k and F_z at 9.8 mm/s (n=6); (e) the μ_k as a function of the value of $v\eta/P$, where η is viscosity of PBS, a value of 1 mPa·s was used at room temperature; P is the contact pressure. Wilcoxon signed-rank test, * P< 0.05.

Supplementary information 6: contact area and contact pressure

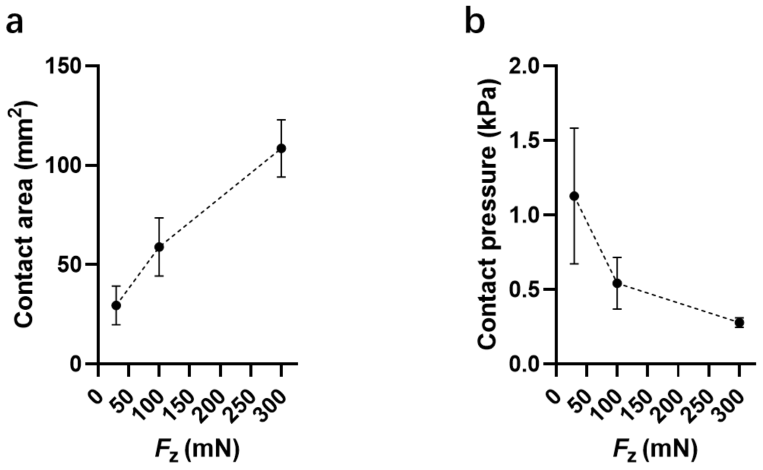

Figure S5. (a)contact area and (b)contact pressure under different F_z

Supplementary information 7: Wilcoxon signed-rank test

Table S2:

	Comparison	n (biological replicates)	P value	Effect size (r)	bootstrap 95% CI for r
μ_s	30 vs 100 mN	6	0.2188	0.56	[0.04, 0.90]
	100 vs 300 mN	6	0.4375	0.39	[0.04, 0.90]
μ_k	30 vs 100 mN	6	0.0312*	0.9	[0.90, 0.90]
	100 vs 300 mN	5	0.8125	0.18	[0.06, 0.90]
μ_s	1.2 vs 2.4 mm/s	4	0.625	0.37	[0.00, 0.91]
	2.4 vs 4.9 mm/s	4	0.875	0.18	[0.18, 0.91]
	4.9 vs 9.8 mm/s	4	0.125	0.91	[0.91, 0.91]

	1.2 vs 2.4 mm/s	3	0.25	0.93	[0.93, 0.93]
μ_k	2.4 vs 4.9 mm/s	3	>0.99	0	[0.00, 0.93]
	4.9 vs 9.8 mm/s	3	0.5	0.62	[0.00, 0.93]

Supplementary information 8: Stribeck Number Calculation

Table S3: Comparison of Stribeck number between *in vivo* blink to *in vitro* Natural Swing experiments

Parameter	Natural Blink	Eyelid-eyeball friction during Natural Swing
**Sliding Speed (mm/s)**	134 [2]	9.8
**Tear Viscosity at the sliding speed (mPa.s)**	1	1
**Contact Pressure (kPa)**	1 [16]	1.2 (Fig. S5)
**Stribeck Number**	1.3×10^{-7}	8.2×10^{-9}

Reference:

1. Chen, Y., et al., *Importance of tribo-pairs to mimic blinking in vitro for dry eye disease research.* Friction, 2026.
2. Kwon, K.-A., et al., *High-speed camera characterization of voluntary eye blinking kinematics.* Journal of the Royal Society Interface, 2013. **10**(85): p. 20130227.
3. Schmidt, T.A., et al., *Transcription, translation, and function of lubricin, a boundary lubricant, at the ocular surface.* JAMA Ophthalmol, 2013. **131**(6): p. 766–76.
4. Björling, M., *Friction in elasto hydrodynamically lubricated contacts: the influence of speed and slide to roll ratio*. 2011, Luleå tekniska universitet.
5. Golebiowski, B., et al., *Lid margins: sensitivity, staining, meibomian gland dysfunction, and symptoms.* Optometry and Vision Science, 2012. **89**(10): p. 1443–1449.
6. Baumli, P., et al., *The Role of Membrane-Tethered Mucins in Axial Epithelial Adhesion in Controlled Normal Stress Environments.* Advanced Biology, 2023. **7**(8): p. 2300043.
7. An, J., et al., *Comparison of a Brush-with-Anchor and a Train-of-Brushes Mucin on Poly(methyl methacrylate) Surfaces: Adsorption, Surface Forces, and Friction.* Biomacromolecules, 2014. **15**(4): p. 1515–1525.
8. Zhang, J., et al., *Recent advances in ocular lubrication.* Friction, 2024. **12**(9): p. 1929–1954.
9. Gipson, I.K., *Distribution of mucins at the ocular surface.* Exp Eye Res, 2004. **78**(3): p. 379–88.
10. Argüeso, P., *Human ocular mucins: The endowed guardians of sight.* Advanced Drug Delivery Reviews, 2022. **180**: p. 114074.
11. Martinez-Carrasco, R., P. Argueso, and M.E. Fini, *Membrane-associated mucins of the human ocular surface in health and disease.* Ocul Surf, 2021. **21**: p. 313–330.
12. Müller, M.T., et al., *Lubrication Properties of a Brushlike Copolymer as a Function of the Amount of Solvent Absorbed within the Brush.* Macromolecules, 2005. **38**(13): p. 5706–5713.
13. Pult, H., et al., *Spontaneous Blinking from a Tribological Viewpoint.* Ocul Surf, 2015. **13**(3): p. 236–49.
14. Pult, H., et al., *Spontaneous Blinking from a Tribological Viewpoint.* The Ocular Surface, 2015. **13**(3): p. 236–249.
15. Gipson, I.K., Y. Hori, and P. Argüeso, *Character of Ocular Surface Mucins and Their Alteration in Dry Eye Disease.* The Ocular Surface, 2004. **2**(2): p. 131–148.
16. Shaw, A.J., et al., *Eyelid pressure and contact with the ocular surface.* Invest Ophthalmol Vis Sci, 2010. **51**(4): p. 1911–7.
